# A High Burden of Infectious Tuberculosis Cases Among Older Children and Young Adolescents of the Female Gender in Ethiopia

**DOI:** 10.3390/tropicalmed10030079

**Published:** 2025-03-17

**Authors:** Zewdu Dememew, Atakilt Deribew, Amtatachew Zegeye, Taye Janfa, Teshager Kegne, Yohannes Alemayehu, Asfawosen Gebreyohannes, Sidhartha Deka, Pedro Suarez, Daniel Datiko, Dan Schwarz

**Affiliations:** 1 USAID Eliminate TB Project, Management Sciences for Health, Addis Ababa P.O. Box 1157, Ethiopia; aabraha@msh.org (A.D.); tworku@msh.org (T.K.); yalemayehu@msh.org (Y.A.); agebreyohan-seconded@msh.org (A.G.); dgemechu@msh.org (D.D.); 2 National TB, Leprosy and Other Lung Diseases Program, Addis Ababa P.O. Box 1234, Ethiopia; amtatachewm@moh.gov.et (A.Z.); taye.letta@moh.gov.et (T.J.); 3 Management Sciences for Health, Global Health Systems Innovation, Arlington, VA 22203, USA; sdeka@msh.org (S.D.); psuarez@msh.org (P.S.); dschwarz@msh.org (D.S.)

**Keywords:** tuberculosis, adolescent, children and adolescents, TB and gender, Ethiopia

## Abstract

The study was conducted in all regions of Ethiopia, except Tigray. It describes types of Tuberculosis (TB) based on gender, age, region, HIV status, and geographic setting in Ethiopia. It is a cross-sectional study that utilized the Ministry of Health’s District Health Information System-based reporting to analyze all types of TB from July 2022 to March 2024. In total, 290,450 TB cases were detected: 42.6% (123,871) were female, 9.4% (27,160) were children (under 15 years of age), and 14.5% (42,228) were adolescents (10–19 years of age). About 48% (20,185) of adolescent TB cases were bacteriologically confirmed, of which 47.5% were females. Compared to children <5 years, the male-to-female ratio is 26% higher among older children (5–9 years of age) (Adjusted Odds Ratio (AOR): 1.26, 95% Confidence Interval (CI): 0.51–2.01)) and 53% higher among adolescents (AOR: 1.53, 95% CI 0.87–2.18). In short, about half of TB cases are infectious among older children and young adolescents of the female gender in Ethiopia. TB among these age categories may be addressed through the integration of TB services with reproductive health services and youth-friendly and pediatric clinics.

## 1. Introduction

The United Nations High-Level Meeting (UNHLM) declaration on tuberculosis (TB) includes global targets to accelerate action to prevent and treat TB among children and adolescents [1]. However, adolescent TB was a neglected area until a road map for fighting TB among children (<15 years) and adolescents (10–19 years) globally was released in 2018 [1], and a similar roadmap focusing on Ethiopia specifically was announced in 2019 [2]. Nevertheless, adolescents have their own peculiar characteristics that expose them to TB, including homelessness, incarceration, and substance use [3]. Also, the Ethiopian Health Management Information System (HMIS) started capturing routine data disaggregated for children and adolescents in 2021.

As they are transitioning between child and adult health services, adolescents face specific age-related challenges in accessing appropriate care in high-burden settings for TB, which could be due to the absence of dedicated adolescent health services that include TB [4,5]. Evidence has shown that adolescents have a high prevalence of TB [6] and exhibit twice the lost-to-follow-up rate of adults (RR 2.0, 95%CI 1.1–3.7), especially for those with HIV infection (23% vs. 6%) [7]. Adolescents are also a recognized key population in the global human immunodeficiency virus (HIV) epidemic, and HIV infection can make them susceptible to developing TB disease [8].

A simple male-to-female TB disease disaggregation not considering different age categories, including adolescents, could be misleading. That is why the proportion of males with notified TB cases is reported to be higher than females in Ethiopia. In fact, TB could be common among the male gender due to the economic and access factors inherent in the male-dominated society, where men have more power than women and some level of privileged access to health care [9]. Therefore, the disaggregation of gender-based TB in different age categories could be important for TB prevention and control, as risk factors could vary depending on both age and gender [10,11]. Specifically, TB among adolescents remains neglected insofar as the characteristics of TB among this age group have not been well described or understood, and interventions have not been designed to address their specific needs. For instance, routine national data failed to capture the 10–19 year age category based on the types of TB before 2021 in Ethiopia [6,7].

Few interventions target TB among adolescents in Ethiopia, and those that do, such as school screening campaigns, tend to be erratic. Despite these, school children and teenagers (10–19 years) usually interact with one another and therefore are easily exposed to TB infection [12,13,14]. Also, little has been described regarding the implications of age and gender for the surveillance and response to TB programs in Ethiopia. Therefore, this study aims to characterize the types of TB in Ethiopia based on gender, age (including adolescents), and geographic setting, to inform future policy and service delivery improvements to better support these communities.

## 2. Materials and Methods

### 2.1. Settings

Ethiopia is the second most populous country in Africa, with adolescents comprising 24% of its total population [15]. The country also has relatively high TB rates, with respective incidence and mortality estimates of 126 and 17 per 100,000 population in 2022. In Ethiopia, 4% of TB cases are also HIV-infected [9], while HIV prevalence in the general population is 0.9% and is higher among women (1.2%) than men (0.6%) [16]. This study was conducted in all regions of Ethiopia, except Tigray. The DHIS2 reporting system was interrupted due to conflict in Tigray so TB data were not available from this region.

### 2.2. Interventions

Led by Management Sciences for Health, the USAID Eliminate TB Project has advocated TB prevention and reduction among adolescents since 2021. The project collaborated with the National TB Program to include the adolescent age category (10–19 years) in the TB program reporting. The national TB guidelines, comprehensive TB/HIV training manuals, and monitoring and evaluation training materials were revised to include childhood and adolescent TB. The Eliminate TB Project has also supported the national TB program in building the capacity of health care personnel working in pediatrics and TB clinics on childhood and adolescent TB. In summary, the adolescent age category has been part of TB program intervention and the TB data reporting system since July 2021.

### 2.3. Data Source

Ethiopia’s Ministry of Health (MoH) utilizes the District Health Information System 2 (DHIS2) for TB data reporting. Data from July 2022 to March 2024 were collected from this system, categorized by TB type, age, gender, region, HIV status, and geographic setting. DHIS2 is a well-established and mature system that has been in use in Ethiopia since 2019. It facilitates data entry at the health facility levels and covers all 4144 health facilities providing TB diagnosis and treatment. Data entered at the health facility level cannot be altered at district, zonal, regional, or federal levels. Data quality and program performance were monitored by a dashboard to provide monthly feedback to health facilities on data accuracy and program effectiveness.

### 2.4. Data Quality

Lot quality assurance sampling (LQAS) and quarterly routine data quality assurance (RDQA) are used in health facilities and districts in the country to ensure data quality. LQAS is used to assess whether the desired level of accuracy has been achieved by comparing data in TB unit registers and the DHIS2 TB reports at the health facility level. RDQA checks the level of over or under reporting (acceptable reporting level between +5 and −5%) comparing TB data in the TB unit register to the data entered in DHIS2 and reported. Also, each reporting health facility verifies data completeness and consistency before submission to the next administrative level.

### 2.5. Data Analysis

The male-to-female ratio was described among age categories of 5 year intervals (up until ≥65 years), HIV-positive status, types of TB (extra-pulmonary TB [EPTB], clinically diagnosed pulmonary TB [clinical PTB], and bacteriologically confirmed pulmonary TB [BCPTB]), and geographic categories (among agrarian, pastoralist, and city administrations of the country) using error bar graphs of SPSS version 25. In addition, bivariate and multivariable logistics analyses were undertaken for the male-to-female ratio of TB based on age categories (<5 years, 5–9 years, 10–19 years, and ≥20 years), types of TB, regions in Ethiopia, and geographic categories using STATA version 17. All variables with a *p*-value of less than 0.05 in the bivariate analysis were entered into the multivariable regression. A two-sample proportion test was applied to compare the BCPTB among adolescents and adults.

### 2.6. Ethical Considerations

The Ethics Review Committee (IRB) of Oromia Regional Health Bureau (RHB) reviewed and approved the study protocol using the routine DHIS2 (reference number of BF/HBOR/1512). The IRB issued a waiver to use TB patients’ data as the DHIS2 contains aggregate data without personal identifying information.

## 3. Results

### Sociodemographic and Clinical Characteristics

During the period of July 2022–March 2024, 290,450 TB cases were detected in Ethiopia—42.6% (123,871) among females and 9.4% (27,160) among children under 15 years of age. The range for the proportion of TB among females was 20.7%; 52.5% in the 10–14 years and 31.8% in the ≥65 years age category. Young adolescents in the 10–14 years age group have the highest proportion of TB among females at 52.5%.

Of TB patients with HIV infections (TB/HIV co-infection), 52.5% were females. Also, females comprised 55.3% of TB/HIV co-infected older children and young adolescents (5–14 years), and 52.9% of TB/HIV co-infected adults above 15 years of age.

Overall, adolescents accounted for 14.5% (42,228) of all TB cases, and the proportion of BCPTB among them was 47.8% (20,185). Of BCPTB adolescents, 47.3% were females, whereas females comprised only 44% of all TB patients in general. Pastoralist regions (Benishangul Gumuz, Afar, and Gambella) have higher proportions of TB cases among males (>60%) (Table 1).

For BCPTB cases, the ratio between males and females is less than one (error bar below one) in children 10–14 years of age (or young adolescents), which shows that BCPTB is dominant among females in this age category. Nevertheless, the ratio is greater than one and steadily increasing for the age categories of those older than 15 years (error bar is entirely above one), indicating that BCPTB is prevalent among male adults. However, the error bar for BCPTB touches or crosses one in the 0–9 age groups, showing there is no difference in the proportions of TB among males or females in this age category.

In EPTB, the ratio between males and females was around one (error bar touches or crosses one), except in the age category of 0–4 years. This means that more under-five male children are diagnosed with EPTB as compared to their female counterparts. There was no significant gender-based difference in EPTB in the remaining age categories.

**Figure 1:**
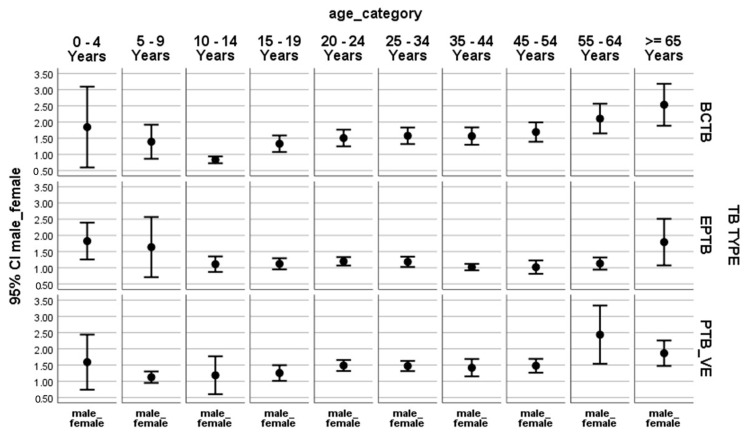
Male-to-female ratio by age and types of TB in Ethiopia, July 2022–March 2024: TB TYPE: type of tuberculosis; BCPTB: bacteriologically confirmed pulmonary tuberculosis; PTB_VE: smear-negative or clinical pulmonary tuberculosis, EPTB: extra-pulmonary tuberculosis; 95% CI: 95% Confidence Interval; male_female: the ratio of males to females; age_category: categories of ages in years.

Among clinically diagnosed PTB patients (PTB_ve), the ratio between males and females, or the error bar, is more than one in adults and the bar touches or crosses one in children (0–14 years). That is, the magnitude of clinically diagnosed PTB is common among adult males, while there is no significant difference in children based on gender (Figure 1).

In the agrarian regions (where 75% of the population makes their living through farming, i.e., Oromia, Amhara, SNNPR, and Tigray), the ratio between males and females is greater than one until 5 years of age, drops below one thereafter, moves back steadily to one in the 10–14 years group, and increased to be equal or greater than one for the groups older than 19 years of age; that is, except in the age group of 5–14 years, where there is no gender-based difference, TB is more prevalent among males in agrarian regions.

Similar to the overall TB at national level, there is no difference in the proportion of TB between males and females (error bar touches or crosses one) in pastoralist regions, except in children <5 years of age, where more males have TB.

**Figure 2:**
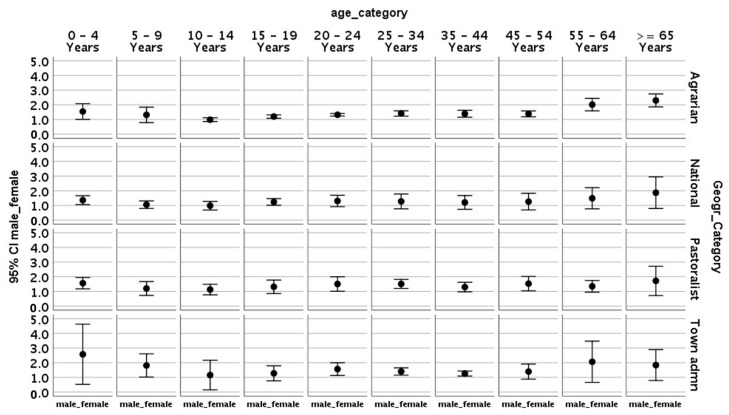
The male-to-female ratio in error chart by age and geographic category in Ethiopia: for each age category, if the upper and lower borders of error bars are below one, the proportion of TB among females is significantly higher than that of males, and the reverse is true if the error bar graph is entirely above one. If the bar graph touches or crosses one, there is no statistically significant difference in the proportion of those with TB among males and females in the specified age category.

However, in city or town administrations, the ratio between males and females is greater than one in the age group of 20–24 years (TB is common among males), and there is no difference in the remaining age category as the ratio touches or crosses one (Figure 2).

**Figure 3:**
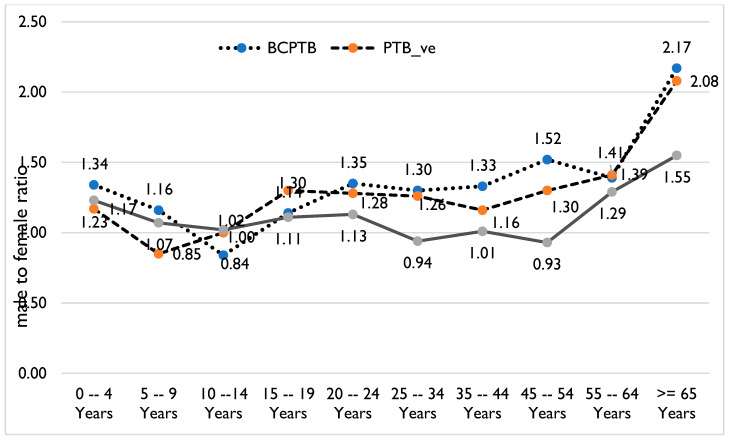
Male-to-female ratio by age category and type of TB, July 2022–March 2024. (BCPTB: bacteriologically confirmed PTB; PTB_ve: clinical PTB or smear-negative PTB; EPTB: Extrapulmonary TB).

Both bivariate and multivariable analyses indicated that the ratio between males and females is associated with age and types of TB. Compared to children <5 years of age, the ratio between males and females is 26% greater among children aged 5–9 years (AOR 1.26, 95% CI 0.51–2.01) and 53% greater among those 10–19 years of age (AOR 1.53, 95% CI 0.87–2.18). EPTB cases are higher among males in children <10 years of age, while BCPTB cases are higher among females in children 10–14 years of age. Also, EPTB is equal or more prevalent among females 34–54 years of age (*p*-value < 0.001) than among males in the same age category (Table 2a,b, and Figure 3).

The 47.8% proportion of BCPTB cases among adolescents is greater than the 44.7% proportion of BCPTB among all age groups (Z-score = 7.5, *p* value = 0.0001). Also, the proportion of female BCPTB patients among adolescents (47.3%) is greater than the proportion of female BCPTB patients (44%) among all TB patients (Z-score = 5.0; *p* value <0.0001). The difference in the proportion of female BCPTB patients among adolescents and the proportion among all age groups is also statistically significant in the Sidama, Oromia, and South Ethiopia regions (Z-score = 4.1–7.8, *p*-value < 0.01), but there is no statistically significant difference in other regions (Z-score < 1.6, *p*-value > 0.181).

## 4. Discussion

Ethiopia is on the World Health Organization’s list of 30 high TB burden countries—third in Africa and eighth in the world—and reports a high proportion of TB cases among males [9]. However, our findings suggest that gender-based TB analysis should consider different age categories to allow for a better understanding of age and gender TB epidemiology. Unlike with all forms of TB cases in adults, BCPTB cases are higher among females than males among older children and young adolescents in Ethiopia.

The higher TB burden among adult males, as compared to females in the same age group, could be due to a greater prevalence of TB risk factors among those men, such as silicosis, imprisonment, alcohol use, deprivation, HIV, cancer, and smoking [11]. A social networking study tried to explain the predominant TB among males, looking at whether distinct mixing patterns by sex and TB disease indicated that TB cases have proportionally more adult male contacts [17]. The higher TB burden among males might also be due to their economic advantage in the Ethiopian culture, which might give them easier access to TB diagnostic services. The gender differences related to health-seeking behavior, stigma, domestic responsibility, access to the economy, a lack of patient-centered care, and access to health care could also contribute to the TB burden difference between males and females [18].

Moreover, the lower TB burden among females in the pastoralist regions, a male-dominated society with a nomadic or mobile lifestyle (Afar, Gambella, Benishangul, and Somalia), might indicate a weak TB program in these regions [19]. Weak health systems in pastoralist regions could hinder efforts for TB care and control [19,20] as passive TB case finding may miss a relatively high number of TB cases among females that cannot easily visit health facilities for TB diagnosis. This could be due to different socioeconomic and cultural factors that might lead to barriers in accessing health care, leaving behind unnotified TB cases in women [21]. The engagement of girls and women in the health extension program in Ethiopia [22] could be a good opportunity to empower them to address barriers to accessing TB diagnosis and treatment service.

Like in our study, findings from Zimbabwe explained that the EPTB rate was lower among reproductive-age males compared to their female counterparts, this mainly ascribed to high HIV infection among females [23]. Studies from Asia also found that while TB of all types was reportedly more prevalent in males, a higher preponderance of EPTB was observed in females [24]. EPTB can lead to pregnancy complications in women, and it warrants surveillance and advocacy for enhancing the development of new diagnostics and new drugs that consider the special needs of women—specifically those living with HIV and those who are pregnant and lactating [25].

The persistent age-related trend in immunologic susceptibility among adolescents and young adults [26] might explain the increasing TB burden among older children, young adolescents, and also adults in our study. That is, puberty is associated with changes in immunity that may contribute to an increased risk of progression to TB disease among these age groups [26,27,28]. Moreover, our study indicated a shift from the less transmissible TB (EPTB and clinical PTB) among children <5 years old to highly transmissible forms of TB (bacteriologically confirmed PTB) among older children and adolescents, which could also be attributable to age-related immunologic susceptibility [3]. Moreover, latent TB infections among children could express themselves as TB disease during adolescence for those affected by malnutrition and HIV [3,29]. Therefore, TB prevention and control activities could consider the age shift for the different types of TB in Ethiopia.

Specifically, older children and adolescents (10–19 years) are highly affected by infectious TB diseases. This could be partly attributable to the fact that adolescents usually spend their time in school and can easily be exposed to TB transmission [13,14]. It could also be due to health challenges associated with pregnancy and childbirth, which may increase the risk of developing TB [30,31]. Hence, older children and adolescents could be a focus area for actively detecting TB cases, which ought to be linked to TB infection control in settings where adolescents are greater in number, such as schools, colleges, and universities.

BCPTB cases were not only higher among adolescents, they were also higher among female adolescents. That is, about half of TB cases among adolescents were infectious cases, as were 47% among female adolescents in Ethiopia. This might be due to late presentation, higher transmission, and low infection prevention and TB prevention services among these groups. Our study is in line with studies that confirmed adolescent females are more susceptible than males to TB [32], TB risk for females increases around the time of menarche, and females have a higher risk of disease progression when compared to age-matched male adolescents [10,26,27]. The study has also shown that an increased BCPTB risk for females relative to males appears to peak at mid-adolescence (10–14 years), which is similar to the results of a recent pooled analysis from high-income countries [10]. These results could be attributable to higher vulnerability to HIV among female adolescents [3,26,27], but the real reason has yet to be explored in Ethiopia. However, TB screening could be integrated into reproductive health services in schools to deal with the TB epidemic among younger girls.

This is the first study that analyzed a huge amount of TB program data at the national level to indicate a gray area regarding TB among adolescent females in Ethiopia. However, the findings should be interpreted cautiously as the study used aggregate routine DHIS2 data. The lack of patient-level data therefore means risk factors for TB could not be exhaustively analyzed. Furthermore, data from Tigray were not part of this study as DHIS2 was interrupted in the region due to conflict.

## 5. Conclusions

The higher proportion of infectious TB cases among school-age children, older children, and young adolescents that are females could necessitate high-quality TB services that are accessible and acceptable to facilitate timely diagnosis and support medication adherence and treatment completion for the early control of TB transmission. TB among female adolescents may be addressed through its integration into reproductive health and youth-friendly and pediatric clinics. In addition, HEWs could consider conducting TB screening of female adolescents at religious and school settings.

## Figures and Tables

**Table 1:** TB cases in Ethiopia by age, region, types of TB, and HIV status, July 2022–March 2024.

Variables	Total New TB ^#^ Cases	Male	% Male	Female	% Female
**New TB cases**	282,979	162,197	57.3	123,970	42.7
**Relapse TB cases**	7471	4354	58.3	3117	41.7
**All TB cases (new and relapse TB cases)**	290,450	166,479	57.3	127,087	42.7
**TB cases with HIV-positive status and on ART by age category**	13,515	6418	47.5	7097	52.5
0–4 years	303	205	67.7	98	32.3
5–14 years	588	263	44.7	325	55.3
≥15 years	12,624	5950	47.1	6674	52.9
**TB cases by age group**					
0–4 years	7776	4411	56.7	3365	43.3
5–9 years	7781	3981	51.2	3800	48.8
10–14 years	11,603	5508	47.5	6095	52.5
15–19 years	30,625	16,808	54.9	138,817	45.1
20–24 years	50,152	28,624	57.1	21,528	42.9
25–34 years	73,498	42,380	57.7	31,118	42.3
35–44 years	45,376	25,886		19,490	43
45–54 years	27,687	16,271	58.8	11,416	41.2
55–64 years	16,489	10,148	61.5	6341	38.5
≥65 years	11,992	8180	68.2	3812	31.8
BCPTB	108,671	62,333	57.4	46,339	42.6
Clinical PTB	108,671	32,660	57.5	24,176	42.5
EPTB	63,172	33,068	52.3	30,104	47.7
Relapse	7471	4354	58.3	3117	41.7
**TB cases by region**					
Sidama	17,041	10,030	58.9	7011	41.1
Gambella	2447	1564	63.9	883	36.1
Dire Dewa	3078	1824	59.3	1254	40.7
Amhara	36,768	20,653	56.2	16,115	43.8
South West Ethiopia Region (SWEP)	6544	3647	55.7	2897	44.3
Harari	1560	887	56.9	673	43.1
Addis Ababa	15,374	8049	52.4	7325	47.6
Afar	5215	3172	60.8	2043	39.2
Somali	14,654	8172	55.8	6482	44.2
Central Ethiopia Region (CER)	10,395	5723	55.1	4672	44.9
South Ethiopia Region (SER)	16,230	9143	56.3	7087	43.7
Benishangul Gumuz	1805	1130	62.6	675	37.4
Oromia	97,514	54,042	55.4	43,472	44.6

^#^ TB: Tuberculosis; HIV: human immune-deficiency virus; ART: anti-retroviral therapy; BCPTB: bacteriologically confirmed pulmonary tuberculosis; PTB: pulmonary tuberculosis, EPTB: extra-pulmonary tuberculosis.

**Table 2:** (**a**). Crude odds ratio of male-to-female ratio in Ethiopia, July 2022–March 2024. (**b**). Adjusted odds ratio of male-to-female ratio in Ethiopia, July 2022–March 2024.

(**a**)								
**Variables**		COR (for the ratio between males and females)			95% CI			*p*-Value
**Age Category**								
<5 years		1						
5–9 years		−1.151			−1.899	−0.403		0.003
10–19 years		−1.432			−2.087	−0.778		0
Adult >20 years		0.085			−0.481	0.651		0.769
**Type of TB**								
Clinical PTB		1						
BCPTB		0.201			−0.206	0.608		0.333
EPTB		−0.869			−1.283	−0.454		0
**Region**								
City administration		1						
Amhara		−0.263			−0.979	0.453		0.472
Benishangul Gumuz		0.274			−0.442	0.99		0.453
Gambella		−0.103			−0.837	0.632		0.784
National		−0.182			−0.897	0.534		0.619
Southern Nations and Nationality and Peoples Region (SNNPR)		−0.37			−0.912	0.172		0.181
Oromia		−0.146			−0.862	0.57		0.689
Pastoralist		0.107			−0.414	0.629		0.687
Sidama		0.208			−0.508	0.924		0.568
**Geographic Category**								
City administration		1						
Agrarian		−0.102			−0.522	0.319		0.635
National		−0.178			−0.893	0.538		0.627
Pastoralist		0.081			−0.485	0.647		0.779
(**b**)								
Variables	COR (for the ratio * between males and females)	95% CI		*p*-value	AOR (for the ratio between males and females)	95% CI		*p*-Value
**Age Category**								
<5 years	1				1			
5–9 years	−1.151	−1.899	−0.403	0.003	−1.258	−2.007	−0.509	0.001
10–19 years	−1.432	−2.087	−0.778	0	−1.527	−2.182	−0.872	0
Adult >20 years	0.085	−0.481	0.651	0.769	0.013	−0.553	0.579	0.964
**Type of TB**								
Clinical PTB	1				1			
BCPTB	0.201	−0.206	0.608	0.333	0.16	−0.246	0.567	0.439
EPTB	−0.869	−1.283	−0.454	0	−0.928	−1.342	−0.513	0

* The ratio between males and females is the number of TB cases among males divided by that among females to three decimal places. The ratio between males and females, as an outcome or independent variable, was compared in each category of dependent variables (bivariate analysis) using a Crude Odds Ratio (COR), and the ratio between males and females was compared among different variables (multivariable) (those with a significant association showed a *p*-value < 0.05 during bivariate analyses) using an Adjust Odds Ratio (AOR). The association was taken as statistically significant if the 95% confidence interval (CI) did not cross 1 or −1 or the *p*-value was <0.05. PTB stands for pulmonary tuberculosis, BCPTB refers to bacteriologically confirmed PTB, and EPTB reads as extra-PTB.

## Data Availability

All data used here are available in the manuscript.

## Abbreviations

The following abbreviations are used in this manuscript:

BCPTB	Bacteriologically confirmed pulmonary TB
DHIS2	Health District Health Information System 2
EPTB	Extra-pulmonary TB
HIV	Human immunodeficiency virus
LQAS	Lot quality assurance sampling
TB	Tuberculosis
UNHLM	United Nations High-Level Meeting
